# Effectiveness of a home health monitoring and education program for complex chronic patients, led by primary care nurses

**DOI:** 10.3389/fpubh.2023.1281980

**Published:** 2023-11-09

**Authors:** María S. Soldado-Matoses, Jordi Caplliure-Llopis, Carlos Barrios

**Affiliations:** ^1^School of Doctorate, University of Valencia Saint Vincent Martyr, Valencia, Spain; ^2^Department of Health La Ribera, Valencia, Spain; ^3^Department of Nursing, School of Medicine and Health Sciences, Catholic University of Valencia Saint Vincent Martyr, Valencia, Spain; ^4^Institute for Research on Musculoskeletal Disorders, Catholic University of Valencia Saint Vincent Martyr, Valencia, Spain

**Keywords:** nursing home visits, chronic disease, primary health care, health services, older adult, mortality, hospital admissions, emergency care

## Abstract

**Background:**

The challenge of chronicity has led developed countries to design strategies to respond to the new needs of complex chronic patients (CCP). There is evidence supporting better beneficial effects and more efficient care for CCP when home-base care programs are provided by Primary Health Care professionals. The main objective of the present study was to assess the effectiveness of a nursing intervention program of home visits for CCP analyzing the use of health services in terms of hospital admissions, emergency care unit visits, and mortality rate.

**Methods:**

A quasi-experimental study was designed to retrospectively evaluate the effectiveness of a 3-year proactive, individualized nursing intervention in improving health outcomes measured by health service utilization (hospitalization, emergency care, and nursing home visits) in these patients. Of the 344 complex chronic patients participating in the study, 93 were assigned to the intervention group (IG) and 251 to the control group (CG).

**Results:**

Along the period of study, the number of home visits in the IG almost tripled in relation to the CG (14.29 ± 4.49 vs. 4.17 ± 2.68, *p* < 0.001). Admissions in the first and second year of the study period were lower in the intervention group *p* = 0.002 and *p* < 0.001 respectively. All the participants in the control group were admitted at least once during the study period. In contrast, 29.0% of the participants in the intervention group never had a hospital admission during the 3-years study period. The number of ED visits to the emergency department was significantly lower in the IG during the 3 years of the study periods. The cumulative number of emergency visits in the IG was half that in the CG (5.66 ± 4. vs. 11.11 ± 4.45, *p* < 0.001, Cohen’d,1.53). A total of 35.5% of the participants in the intervention group visited the emergency department on three or fewer occasions compared to 98% of the subjects in the control group who visited the emergency department on more than six occasions (*p* < 0.001). The 3-year overall mortality rate was 23.5% in the control group and 21.6% in the nursing home visit program. These differences were not statistically significant.

**Conclusion:**

The program demonstrated its effectiveness in reduction of hospital admissions and visits to the emergency department. The program had no impact on mortality rate. This program of home visits reinforces the role of primary care nurses in advanced competencies in chronicity.

## 1. Introduction

Patients with complex chronic diseases are broadly defined as those having various combined morbidities, implying severe disabilities or functional limitations that require multidisciplinary health care providers (1). In the later stages, there is a reciprocal exacerbation between chronicity and loss of independence, further deteriorating patient well-being. This category of complex chronic patients (CCP) contributes substantially to increased morbidity and mortality (2). The progressive increase in the prevalence of multimorbidity has become a great challenge for healthcare systems in developed countries. The escalating prevalence of multiple concurrent health conditions presents a significant hurdle for healthcare systems in industrialized nations. This upsurge in enduring health problems results in amplified utilization of medical provisions and personnel, consequently leading to elevated healthcare expenditures for governments.

In the last two decades, different strategies to address pluripathology and frailty have been implemented, particularly in CCP (3–5). Many developed countries have allocated health resources to identify this segment of the CCP population and develop responses according to their needs, introducing new specific care models (3, 6). Basically, these models can be grouped into 2 different types: (i) Systemic models, focused on reorienting the health system such as the Chronic Care Model developed by the MacColl Center for Health Care Innovation, and commonly referred to as the Wagner chronic care model (6, 7). These models propose a case management strategy from Primary Care to prevent complications and exacerbations to improve health outcomes and quality of life and to decrease the use of high-cost resources; (ii) population models, which focus on identifying and responding to the needs of chronic patients, such as those of the Kaiser Permanente organization (8). These last models are based on strategies for segmenting the population based on complexity and level of care. Complexity was defined as comorbidity, readmission, polypharmacy, and dependency (9).

In 2012, the Spanish Ministry of Health, Social Services, and Equality published a strategy to approach chronicity in the National Health System and encouraged Autonomous Communities to deploy territorial programs for the care of complex chronic patients. In 2014, the Valencia Regional Government published its own strategy for the care of chronic patients, leaving it up to each Health Department to specify its implementation (10, 11).

To the best of our knowledge, there is little evidence of the effectiveness of new models of chronic patient care. Rather than a reorganization of the care model, the development of these plans has been based, in general, on the use of different instruments (stratification, case management, liaison nursing, home care, telemedicine, etc.) that demonstrate variable and controversial effectiveness and efficiency (12). The evaluation of health service interventions typically involves the assessment of a comprehensive package of services, and it is often challenging to isolate the individual effects of specific components within these interventions (13). Consequently, even in cases where statistically significant effects of interventions are not observed, it is essential to acknowledge that some components may still be beneficial.

Managing complex chronic patients in hospitals imposes significant financial and healthcare system burdens. These patients often incur higher costs (14, 15), experience longer hospital stays, and contribute to readmissions, necessitating significant resource allocation (16–18). Addressing these challenges is crucial for maintaining the financial sustainability of healthcare systems and ensuring high-quality care for complex chronic patients. A comprehensive report by The Commonwealth Fund in 202, titled “United States Health Care from a Global Perspective, 2022: Accelerating Spending, Worsening Outcome” emphasized that the management of complex chronic patients requires significant resource allocation, including specialized staff, technology, and care coordination efforts, contributing to the overall financial burden on healthcare systems (19).

In contrast, there is multiple evidence supporting better beneficial effects and more efficient care for CCP when home-base care programs provided by Primary Health Care professionals are designed (20). The main objective of home care interventions is to reduce institutionalized care (e.g., nursing homes and hospitals) (8, 9). Most home-care services are provided by primary care practitioners, geriatricians, nurses, and social workers. However, primary care or community nurses serve as central coordinators of care for complex chronic patients. They can coordinate appointments, medications, and referrals, ensuring that patients receive timely and appropriate care. Nursing home care visits should include not only health assessments focused on treating observed problems but also health education and social and psychological support (21–24). In their home visits, primary care nurses also play a key role in medication management, ensuring that patients understand and adhere to their medication regimens. Improved adherence can prevent exacerbations and hospitalizations.

To date, nursing interventions that seem to have the greatest impact on the strategy of chronic care in Spain are case management, home care programs from primary care, and telemonitoring (12, 25). Although they demonstrated favorable results in terms of effectiveness and satisfaction, more studies are needed to prove the efficiency of nursing contribution in chronicity. If interventions of home health care for chronicity provided by home nurses obtain better results, it could be asked whether the most efficient plan would be to explicitly strengthen the figure of the home nurse with advanced competence in chronicity within the scope of PC.

Aligned with the National strategy for addressing chronicity, the La Ribera Health Department decided to implement it in January 2017, adapting it to its own idiosyncrasies. The program was implemented until March 2020, when nurse home visits were interrupted due to forced confinement related to the first wave of the Sars-CoV2 pandemic. In 2021, the need for reorientation and implementation of home care prompted exploration of the effects of these nursing interventions. In addition, questions arise concerning how that change in the culture of the approach to chronicity was perpetuated in the daily practice of professionals and how it was the current impact on health outcomes for chronic patients.

To address these issues, the main objective of the present study was to retrospectively assess the effectiveness of a proactive intervention program of home visits implemented by community nurses for patients with combined chronic conditions as compared to a control group with similar diseases. This analysis spans a 3-year period, from 2017 to 2019. Effectiveness was assessed in terms of service utilization, including hospital admissions, emergency department (ED) visits, and mortality rates, and home visits conducted by nursing staff.

## 2. Materials and methods

### 2.1. Study design

This was a quasi-experimental study with a control group and non-probabilistic convenience sampling. In January 2017, the total population of Sueca’s health area (Valencia, Spain) was 27,598 inhabitants. The total population was classified by health status and associated comorbidities using the records of the Valencian Community Patient Classification System (SCP-CV), which is based on the International Statistical Classification of Diseases and Related Health Problems and 3 M Clinical Risk Groups tool (CRG) (26, 27). Using established demographic, diagnostic, and procedural information, the CRG classification system categorizes individuals into one of nine primary health status groups. These groups ranged from severe (e.g., individuals with a history of heart transplant) to well (e.g., those with no chronic health issues or other discernible risk factors). Patients included in CRG 6 suffer from chronic disease in two or more organ systems (diabetes mellitus and congestive heart failure for example). Patients with multiple dominant chronic diseases (three or more) such as diabetes mellitus, congestive heart failure, and chronic obstructive pulmonary disease are included in CRG 7. Within each CRG, chronic illnesses and conditions are additionally segmented into six distinct levels of Severity of Illness (SOI).

### 2.2. Inclusion and exclusion criteria

In the current study, the target groups were CRG 6 and CRG 7 patients with severities of 5 (severe illness) and 6 (extremely severe) respectively. Additional inclusion criteria were age over 65 years, following a classic home care at demand, Charlson’s comorbidity status ≥3, and high cost of pharmacy prescriptions (>p95) (28). Individuals in the end-of-life stage (CRG 8–9), those receiving treatment for their conditions through private health insurance companies, and mobile population (individuals who do not reside at their usual residence for more than 3 months per year) were excluded from the study.

### 2.3. Final sample selection

**Figure 1:**
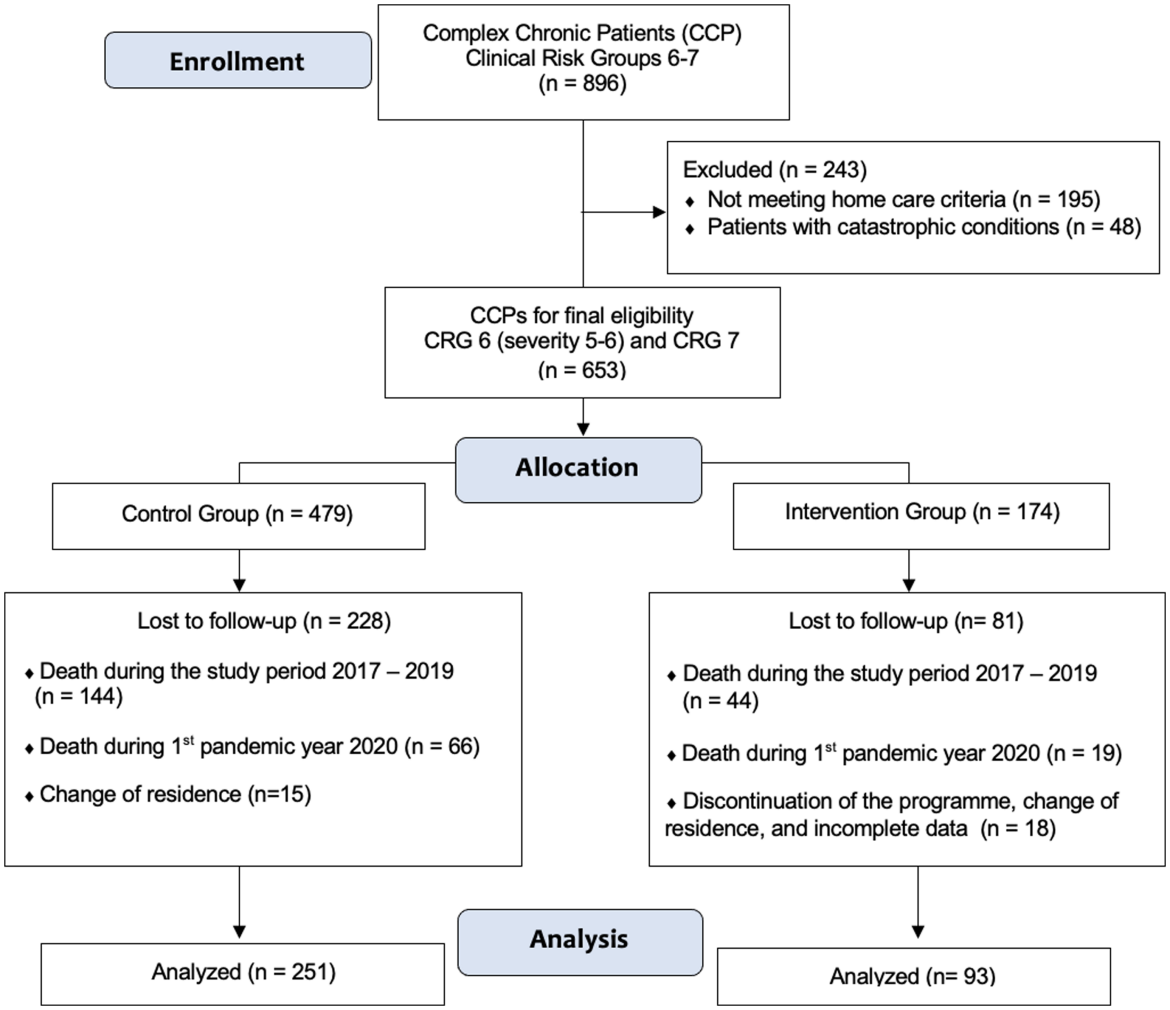
Trial flow chart.

A sample of 653 patients was considered to have CCP included in the CRG 6–7 was finally considered for allocation to the intervention and the control group (Figure 1). Out of these 653 CCP, residents of Health Zone B11 of the La Ribera Health Department (Sueca), 174 (26.6%) were initially selected at random to follow the intervention protocol (IG) in January 2017. Our primary healthcare area is composed by 10 Basic Care Units (BCU), which involve a general practitioner and a nurse responsible for patients aged 15 and above. Each BCU is tasked with delivering care to a designated group of individuals, typically ranging from 1,350 to 1,550 patients. Members of each BCU selected at random at least 20 individuals from their pool of CCPs.

The remaining 479 CCPs were considered as the control group (CG). At the time to retrospectively evaluate the sample in 2021, and excluding patients with incomplete data, those lost because of changes in the healthcare area or discontinuity of the nurse home visits program, a sample of 93 alive patients could be finally analyzed in the IG (Figure 1). In this IG, 44 patients died during the period of study, and other 19 died during the first pandemic year, just before analyze data. Among the 479 patients who did not receive programmed nursing home visits and followed the usual care when they ask for home visit, data were finally recorded from 251 subjects who formed the CG. Among control patients, 144 died during the period 2017–2019, other 66 in 2020, the first pandemic year (Figure 1).

### 2.4. Sample size

Even though the present study is based on a non-probabilistic convenience sampling method, the sample size was calculated to ensure its representativeness. The sample size was estimated to detect a difference between two means using “annual hospital admissions” as the most relevant variable and considering a difference greater than 1 unit as clinically relevant. Accepting an alpha risk of 0.05 and a beta risk of 0.2 in a two-tailed test, we require 67 subjects in the first group and 180 in the second group to detect a difference equal to or greater than 1 unit. It is assumed that the common standard deviation is 2.35. A follow-up loss rate of 10% has been estimated. A balance factor of 2.7 has been established between the groups. Participants under the current study exceeds sample size calculations and therefore the results obtained could have stronger value.

### 2.5. Intervention

Three years of intervention were considered. The first two-year period was from March 2017 to March 2019. The third year covered from March 2019 to March 2020 when the program was interrupted because of the appearance of the first wave of the Sars-CoV2. Therefore, the first 2 years were considered regular health-care years; the third year reflected the pre-pandemic year. Data from the 3-year intervention period were evaluated in January 2021 just after the last wave of the Sars-CoV2 pandemic.

Active recruitment of patients throughout the database of the health department was performed using the reference nurse key. The intervention consisted of at least 3 structured home visits per year. The main objectives of these visits are summarized in Table 1. During the first visit, the patient’s characteristics and health situation were assessed, and relevant changes were implemented - in consensus with the patient and primary caregiver - after identifying specific problems and needs. The successive visits involved reevaluation of the situation introducing new preventing and therapeutic measures if required. Each visit followed similar methodology involving three stages:

**Table 1:** Intervention objectives of the 3 scheduled structured visits per year.

Perform a comprehensive assessment of the patient, including functional and cognitive assessment, family Apgar, and nutritional assessment using the MNA questionnaire (29)
Establish contact at least within 48 h post admission to the hospital or the emergency department to ensure continuity of care process, maintain care at home, social-health center or residence and prevent readmission.
Training the patient’s motivation and adherence to treatment so that he/she becomes involved in his/her self-care, promoting self-efficacy.
Systematic review of medication
Assessing pain in patients
Prescription of programs that promote patient autonomy: walking exercise, fall prevention, and pressure ulcer prevention.
Promoting healthy eating
More specifically: use of inhalers, weight, insulin administration, and diuretics in congestive heart failure
Early mobilization of resources if needed.

Stage 1 (Person-centered Review)

- Define therapeutic goals aligned with the patient’s life prognosis.
- Empower patients: Involve the patient or primary caregiver in decision-making. Feedback is considered a strong point of the intervention.

Stage 2 (Particular Health Problems)

- Identify the most relevant health problems.
- Determine the patient’s or primary caregiver’s level of knowledge about their most relevant health problem.
- Conduct a functional, cognitive, and social environment assessment:
- Functional (Barthel scale)
- Cognitive (Pfeiffer scale)
- Social (Family Apgar)
- Identify other relevant problems/needs/risks.
- Palliative Needs (NECPAL scale)
- Fall Risk (Downton scale)
- Risk of Pressure Ulcer Development (Norton scale)
- Risk of Malnutrition (MNA scale)
- Systematic medication review.
- Identify the primary caregiver.

Stage 3 (Care giving)

- Monitoring of clinical and analytical parameters.
- Vital signs measurement.
- Blood sample collection at home and determination of INR.
- Planning of scheduled home-based techniques.
- Catheter changes, PEG tube changes, etc.
- Cleaning of subcutaneous reservoirs, central venous catheter cleaning.
- Administration of scheduled medication with a prescribed frequency, by a different route of administration than oral.
- Immunizations: influenza, and pneumococcus vaccination campaigns.
- Symptom control, with special attention to pain.
- Promotion of healthy eating using the “plate method.”
- Encouragement of physical activity.
- Empower self-care: Training in disease management skills if needed.
- Inhaler usage.
- Weight monitoring for heart failure patients and adjusting diuretic doses.

### 2.6. Data analysis

Data were analyzed retrospectively after the intervention in the three different periods of study (2017 and 2018 regular years, and 2019 pre-pandemic year). Data were obtained from the outpatient care information system (SIA) and the information system of hospital admission (Nou-SIS) of the health department. Participation in the assigned group was an independent variable. Other independent variables with modifying effects were patient age, sex, and degree of dependence. The other dependent variables were related to the use of services: hospitalization, emergency care, mortality, and nursing home visits.

For the statistical analysis of results, quantitative variables were described as the mean, standard deviation (SD), and 95% confidence interval (CI). Measures of central tendency were compared using the *t*-test for independent samples or ANOVA. For effect size analysis, Cohen’s d was calculated. Categorical variables were described as frequencies and percentages. These variables were compared using the Chi-square test or Fisher’s exact test. The statistical program used was the IBM SPSS software v22. Statistical significance was set at *p* < 0.05.

### 2.7. Ethical considerations

Since this was a retrospective study, which was performed under the conditions of routine clinical practice, informed consent was not required from the patients. This study was approved by the Ethics Committee for Clinical Research of the Department of Health, La Ribera, Valencian Community, Spain.

## 3. Results

### 3.1. Participants profile

The distribution by sex, age, anthropometric profile, and level of disability is shown in Table 2. The percentages of males and females were very similar in both groups, with 51.0% of males in the control group and 53.8% in the intervention group. The mean age was slightly higher in the intervention group; however, the difference was not statistically significant (*p* = 0.158). Regarding anthropometric characteristics, differences between the two groups were only found in weight, with the mean weight being lower in the intervention group (76.4 ± 13.3 vs. 80.1 ± 12.2, *p* = 0.026). There were no differences between the two groups in the percentage of moderate or severe disability according to the Barthel scale (18.4% in the intervention group vs. 19.9% in the control group). However, mean Barthel scale scores were higher in the intervention group (73.5 ± 18.1 vs. 66.0 ± 18.2, *p* < 0.001). The distribution of participants according to clinical risk groups was almost similar in the control and intervention samples. Most of the patients (87.2% in CG, and 87.1% in IG) were classified into the CRG 6 (2 chronic diseases). The most prevalent chronic diseases in both groups were combination of cardiologic illnesses (congestive heart failure, ischemic cardiopathy), chronic obstructive pulmonary disease, neurodegenerative diseases (Alzheimer and dementias), and diabetes mellitus.

**Table 2:** Distribution by sex, age, anthropometric profile, and level of disability of the sample.

	Control group (*n* = 251)	Intervention group (*n* = 93)	*p* (Cohen’s d)
Males Females	128 (51.0%) 123 (49.0%)	50 (53.8%) 43 (46.2%)	0.648
Age (yr)	78.9 ± 8.6 (77.9–79.97)	80.6 ± 11.3 (78.2–82.8)	0.154 (0.16)
Weight (kg)	80.1 ± 12.2 (78.6–81.6)	76.4 ± 13.3 (73.3–79.5)	0.026 (0.28)
Stature (cm)	161.9 ± 8.1 (160.9–162.9)	160.8 ± 9.0 (158.7–162.9)	0.325 (0.12)
BMI	30.9 ± 5.9 (30.1–31.6)	29.5 ± 4.6 (28.4–30.6)	0.070 (0.26)
Disability (Barthel) Independents Light Moderate Severe	15 (6.0%) 186 (74.1%) 34 (13.5%) 16 (6.4%)	8 (8.6%) 67 (72.0%) 12 (12.9%) 6 (6.5%)	0.059
Barthel score	66.0 ± 18.2 (62.8–67.6)	73.5 ± 18.1 (69.6–77.1)	<0.001 (0.41)
Comorbidities CRG 6 (2 Chronic diseases) CRG 7 (3 Chronic diseases)	219 (87.2%) 81 (87.1%)	32 (12.8%) 12 (12.9%)	0.969

BMI, body mass index; CRG, clinical risk groups.

When the sample was analyzed separately by sex, there were more differences between women in both groups than between men (Table 3). Women in the intervention group were older than those in the control group (*p* = 0.006) and had lower weight and BMI (*p* < 0.001 and *p* = 0.001, respectively). However, there were no differences in the Barthel index scores. Among the men in both groups, the mean age did not show significant differences, and the only difference in anthropometric measures was a slightly lower mean height in the intervention group. In contrast to the women, there were statistically significant differences between the two groups of men in the mean Barthel index score, which was lower in the control group (63.3 ± 18.6 vs. 79.0 ± 15.4, *p* < 0.001).

**Table 3:** Characteristics of the sample depending on the sex of the participants.

	Males			Females		
	Control (*n* = 128)	Intervention (*n* = 50)	*p* (Cohen’s d)	Control (*n* = 123)	Intervention (*n* = 43)	*p* (Cohen’s d)
Age (yr)	80.0 ± 7.0 (78.6–81.0)	78.6 ± 12.2 (66.8–83.0)	0.360 (0.14)	77.9 ± 9.8 (76.1–79-6)	82.8 ± 10.0 (79.6–85.7)	0.006 (0.49)
Weight (kg)	79.2 ± 12.6 (76.9–81.3)	79.6 ± 11.8 (75.9–83.2)	0.835 (0.03)	81.1 ± 11.8 (78.9–83.2)	72.0 ± 14.3 (66.7–77.25)	<0.001 (0.69)
Stature (cm)	168.7 ± 3.7 (168.0–169.3)	166.2 ± 6.9 (164.1–168.3)	0.004 (0.45)	154.5 ± 4.5 (153.7–155.4)	153.4 ± 5.8 (151.2–155.5)	0.228 (0.21)
BMI	27.8 ± 4.5 (27.0–28.6)	28.8 ± 3.7 (27.6–28.6)	0.218 (0.24)	34.1 ± 5.4 (33.1–35.1)	30.5 ± 5.5 (28.5–32.5)	0.001 (0.66)
Barthel score	63.3 ± 18.6 (50.02–65.82)	79.0 ± 15.4 (75.62–83.96)	<0.001 (2.56)	68.9 ± 17.3 (64.80–71.50)	67.1 ± 19.0 (61.01–72.8)	0.558 (0.09)

### 3.2. Nurse home visits

Table 4 shows the number of nursing home visits to the participants in the control and intervention groups. In the latter group, as required by the intervention, the total number of visits to the participants almost tripled in relation to the control group (14.29 ± 4.49 vs. 4.17 ± 2.68, *p* < 0.001). In the intervention group, a minimum of two and a maximum of 18 visits were performed during the entire period, with a 95% confidence interval between 13.77 and 15.43. In this group, 66% of the participants received more than 12 visits during the 3-year period. In the control group, the confidence interval for the mean of total home visits was 3.84–4.51 visits. In this group, 12.5% of the participants received only a single nursing home visit during the entire period, and 87.1% received a maximum of two home visits per year.

**Table 4:** Mean number of nursing home visits to participants in each group and number of hospital admissions and emergency room visits during the study periods.

	Control (*n* = 251)	Intervention (*n* = 93)	
Nurse home visits	Mean ± SD (95% CI)	Mean ± SD (95% CI)	p (Cohen’s d)
total cumulative	4.17 ± 2.68 (3.84–4.51)	14.29 ± 4.49 (13.77–15.43)	<0.001 (2.73)
visits / year	1.39 ± 0.89 (1.28–1.50)	4.76 ± 1.49 (4.59–5.14)	<0.001 (2.74)
Hospital admissions			
1st year (2017)	0.86 ± 0.95 (0.75–0.99)	0.50 ± 0.95 (0.31–0.70)	0.002 (0.37)
2nd year (2018)	2.00 ± 0.75 (1.89–2.08)	0.61 ± 0.74 (0.46–0.77)	<0.001 (1.86)
3rd year (pre pandemic 2019)	0.76 ± 0.77 (0.65–0.84)	0.91 ± 1.57 (0.57–1.22)	0.245 (0.12)
Total admissions	3.63 ± 1.53 (3.42–3.80)	2.02 ± 2.16 (1.58–2.48)	<0.001 (0.86)
Emergency department visits			
1st year (2017)	2.89 ± 1.71 (2.67–3.10)	1.08 ± 1.74 (0.73–1.46)	<0.001 (1.04)
2nd year (2018)	5.25 ± 1.49 (5.06–5.44)	2.74 ± 1.56 (2.41–3.05)	<0.001 (1.64)
3rd year (pre pandemic 2019)	2.98 ± 1.35 (2.80–3.14)	1.64 ± 2.36 (1.15–2.13)	<0.001 (0.69)
Total ED visits	11.11 ± 3.31 (10.70–11.53)	5.65 ± 4.45 (4.74–6.57)	<0.001 (1.53)

### 3.3. Hospital admissions

Regarding the number of hospital admissions, the intervention group had a lower mean number of admissions during the first year of follow-up (2017/18) compared to the control group (0.50 ± 0.95 vs. 0.86 ± 0.95, *p* = 0.002). The same finding was observed during the second period (2018/19) where a relevant increase of hospital admission was found in the control group (0.61 ± 0.74 vs. 2.00 ± 0.75, *p* < 0.001). However, there was no difference between the groups in the third study period (pre-pandemic year 2019), but not in the total number of admissions in the three periods (Table 4). Considering the whole period of study, the intervention led by nurses showed a large beneficial effect in reducing hospital admissions in CCPs (Cohen’s d: 0.86).

**Figure 2:**
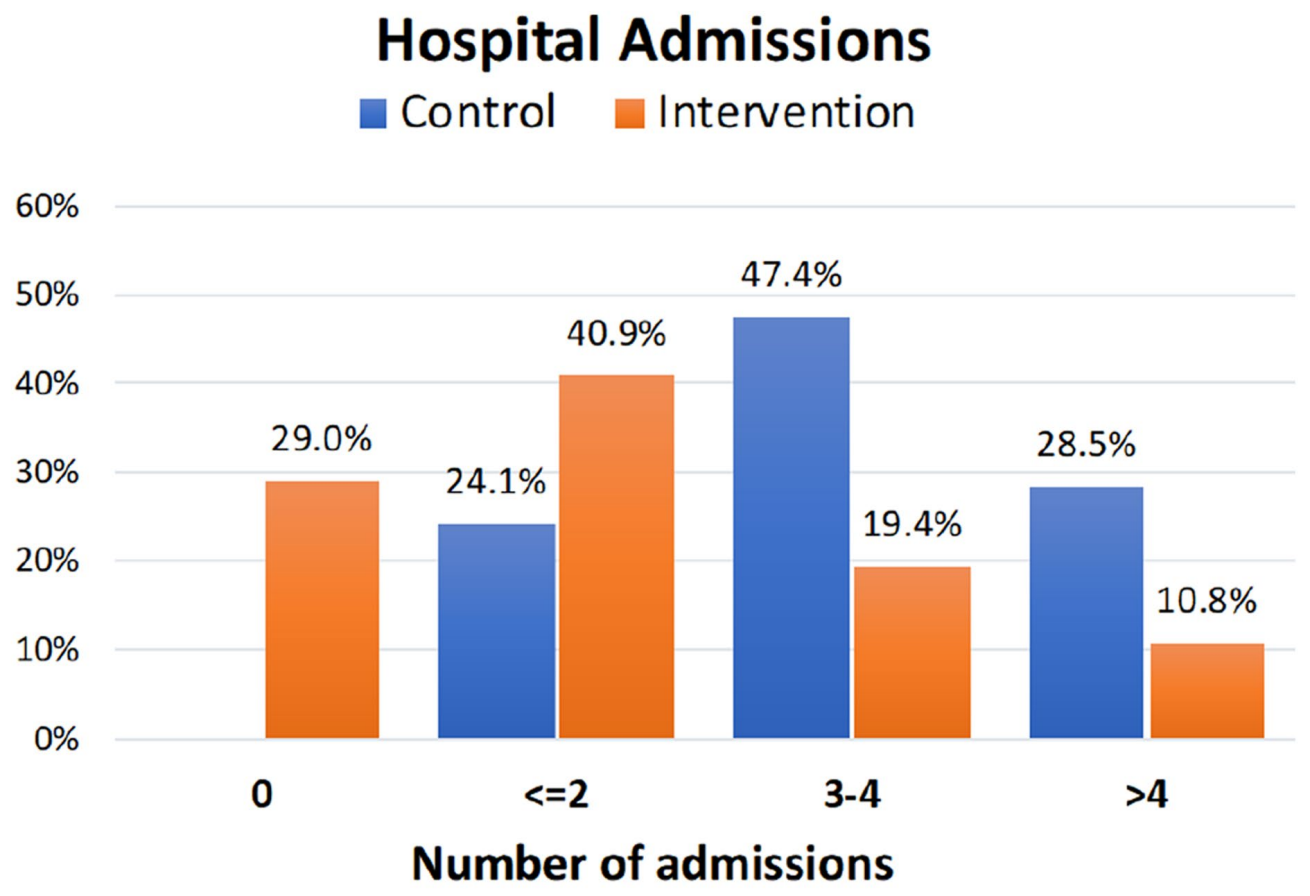
Percentage of cumulative hospital admissions during the study period in the control and intervention groups.

Regarding hospital admission, all participants in the control group were admitted at least once during the study period. On the contrary, a 29.0% of the participants in the intervention group never had a hospital admission during the 3-years study period, and another 40.9% had a maximum of two admissions (Figure 2). In the control group, 75.9% of the participants were admitted to the hospital three or more times, while only 30.2% of the participants in the intervention groups required such a frequency of admissions (*p* < 0.001).

### 3.4. Visits to the emergency department

**Figure 3:**
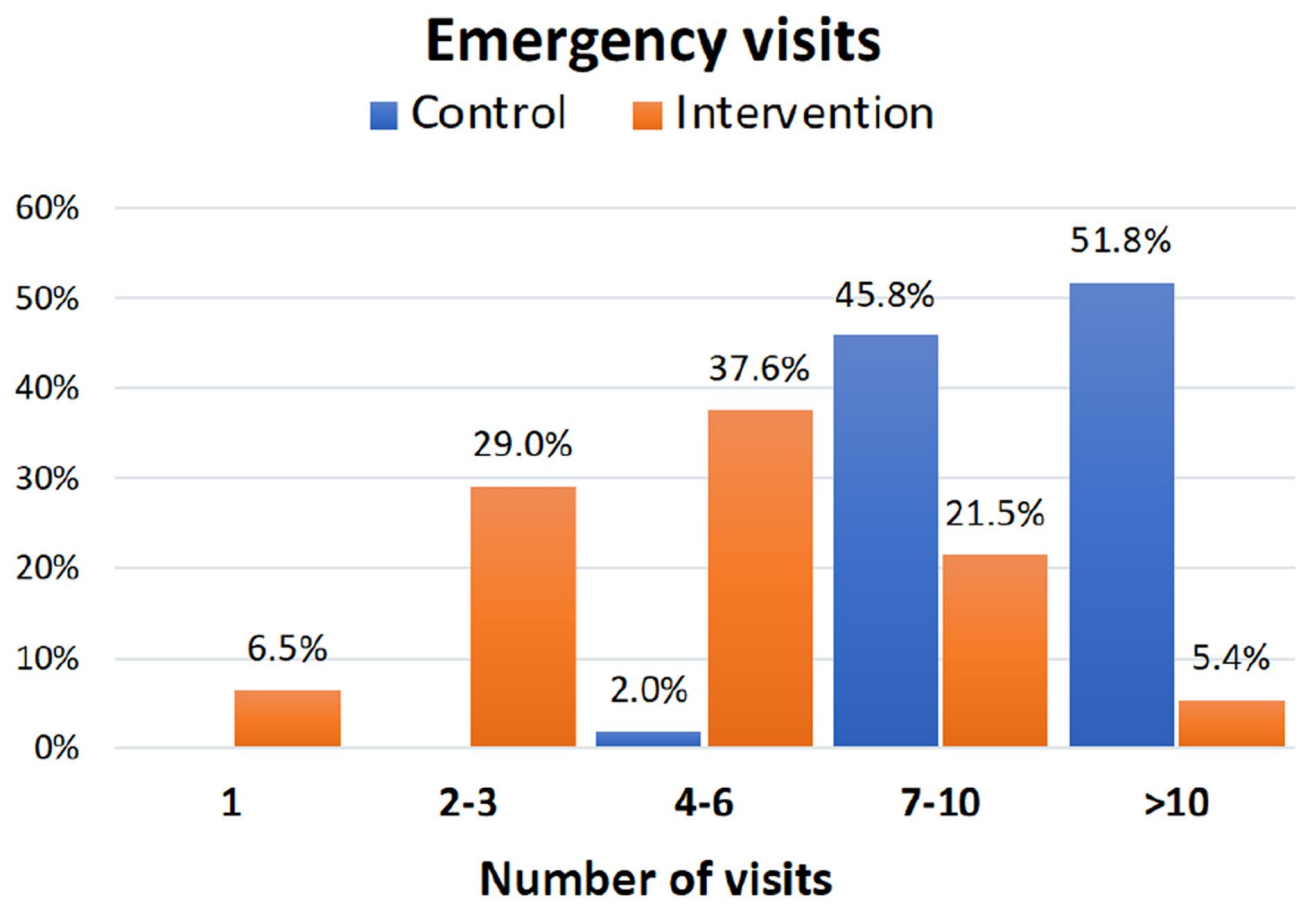
Cumulative percentage of visits to the emergency department during the study period in the control and intervention groups.

Both cumulative visits to the emergency department and those corresponding to the three study periods were approximately 50% less frequent in the intervention group, and the differences were statistically significant in relation to the control group (Table 4). The effect size of the intervention concerning the total number of visits to the ED was large (Cohen’s d: 1.53). A total of 35.5% of the participants in the intervention group visited the emergency department on three or fewer occasions compared to 98% of the subjects in the control group who visited the emergency department on more than six occasions (*p* < 0.001). Figure 3 shows the percentage of participants who visited the emergency room on different occasions. Interestingly, 51.8% of the subjects in the control group visited the Emergency Department on more than 10 occasions throughout the study period compared to 5.4% of the participants in the intervention group (*p* < 0.001).

### 3.5. Impact of sex

When the mean number of hospital admissions and visits to the emergency department during the study period was analyzed by sex and group, it was observed that in both men and women, there were statistically significant differences in the cumulative frequency of hospital admissions and visits to the emergency department, which were higher in men and women in the control group (Table 5). There were no differences between the men and women within both the control and intervention groups. In the women in the intervention group, it was also observed that the mean number of hospital admissions was lower than that of the control group in the first two study periods. The largest effect size of the intervention was found in both the total number of hospital admissions and total visits to the ED (Cohen’s d: 1.29 in males; Cohen’s d: 1.31 in females).

**Table 5:** Mean number of hospital admissions and emergency room visits in the study periods, broken down by sex and group.

	Males			Females		
	Control (*n* = 128)	Intervention (*n* = 50)		Control (*n* = 123)	Intervention (*n* = 43)	
Hospital admissions	Mean ± SD (95% CI)	Mean ± SD (95% CI)	*p*	Mean ± SD (95% CI)	Mean ± SD (95% CI)	*p*
1st year (2017)	0.88 ± 0.98 (0.74–1.08)	0.72 ± 1.16 (0.40–1.09)	0.350 (0.14)	0.85 ± 0.92 (0.67–1.00)	0.25 ± 0.53 (0.08–0.41)	<0.001 (0.79)
2nd year (2018)	1.94 ± 0.77 (1.81–2.07)	0.70 ± 0.76 (0.50–0.95)	<0.001 (1.62)	2.06 ± 0.73 (1.90–2.16)	0.50 ± 0.69 (0.28–0.71)	<0.001 (2.19)
3rd year (pre pandemic 2019)	0.79 ± 0.76 (0.64–0.90)	0.82 ± 1.40 (0.38–1.19)	0.889 (0.02)	0.73 ± 0.79 (0.58–0.87)	1.02 ± 1.76 (0.49–1.55)	0.145 (0.21)
Total admissions	3.62 ± 1–57 (3.35–3.90)	2.24 ± 2.13 (1.64–2.98)	<0.001 (0.73)	3.65 ± 1.49 (3.30–3.86)	1.77 ± 2.18 (1.10–2.43)	<0.001 (1.00)
Emergency department visits						
1st year (2017)	2.88 ± 1.72 (2.61–3.21)	1.02 ± 1.67 (0.57–1.55)	<0.001 (1.09)	2.90 ± 1.71 (2.54–3.17)	1.16 ± 1.85 (0.57–1.59)	<0.001 (0.96)
2nd year (2018)	5.17 ± 1.37 (4.93–5.42)	2.96 ± 1.59 (2.50–3.41)	<0.001 (1.48)	5.32 ± 1.61 (5.04–5.62)	2.50 ± 1.50 (2.04–2.95)	<0.001 (1.81)
3rd year (pre pandemic 2019)	3.06 ± 1.32 (2.80–3.27)	1.58 ± 2.16 (0.95–2.21)	0.002 (0.82)	2.90 ± 1.38 (2.66–3.15)	1.60 ± 2.57 (0.91–2.49)	<0.001 (0.97)
Total ED visits	11.13 ± 3.04 (10.59–11.66)	5.94 ± 4.80 (4.44–6.78)	<0.001 (1.29)	11.08 ± 3.57 (10.46–11.74)	5.34 ± 5.05 (4.10–6.57)	<0.001 (1.31)

### 3.6. Influence of the level of dependence

The results were analyzed according to the participants’ level of dependence (Table 6). In the independent or slightly disabled patient group, statistically significant differences were observed between the participants in the control and intervention groups in the mean cumulative number of visits to the emergency department throughout the study. In the intervention group, these values decreased by more than 50%. There were also differences in the cumulative number of hospital admissions, but no during the third phase of the study, in which the intervention group was significantly higher. In the high- or moderate-dependency groups, the intervention only produced differences in the number of visits to the emergency department during the first and second study periods (*p* = 0.012 and *p* < 0.001, respectively). Considering the whole period of study, the largest effect size of the intervention on the total number of hospital admissions was found in patients with severe/moderate disability (Cohen’s d: 1.22). Concerning total visits to the ED, the largest effect size was found in independent patients and those slight disability (Cohen’s d: 1.76). There were no differences in the mean number of home nursing visits per years between patients with no or slight dependence and those with high/moderate dependence in the intervention group (4.54 ± 2.32 vs. 4.81 ± 1.26, *p* > 0.05).

**Table 6:** Number of admissions and visits to the emergency room in both groups, control and intervention, stratified by level of dependency.

	Independent or slight disability			Severe/moderate disability		
	Control (*n* = 201)	Intervention (*n* = 75)		Control (*n* = 50)	Intervention (*n* = 18)	
Hospital admissions	Mean ± SD (95% CI)	Mean ± SD (95% CI)	*p*	Mean ± SD (95% CI)	Mean ± SD (95% CI)	*p*
1st year (2017)	0.89 ± 0.94 (0.70–0.96)	0.52 ± 0.97 (0.239–0.74)	0.005 (0.38)	1.01 ± 0.96 (0.76–1.27)	0.47 ± 0.87 (0.02–0.92)	0.027 (0.58)
2nd year (2018)	2.00 ± 0.76 (1.90–2.12)	0.74 ± 0.75 (0.58–0.93)	<0.001 (1.65)	1.89 ± 0.65 (1.71–2.06)	0	<0.001
3rd year (pre pandemic 2019)	0.70 ± 0.78 (0.61–0.83)	0.90 ± 1.57 (0.55–1.28)	0.163 (0.16)	0.83 ± 0.78 (0.63–1.04)	0.82 ± 1.59 (0.00–1.64)	0.711 (0.01)
Total admissions	3.60 ± 1.52 (3.36–3.78)	2.17 ± 2.13 (1.70–2.79)	<0.001 (0.77)	3.65 ± 1.64 (3.31–4.19)	1.29 ± 2.17 (0.17–2.41)	<0.001 (1.22)
Emergency department visits						
1st year (2017)	2.90 ± 1.67 (2.62–3.09)	1.00 ± 1.45 (0.66–1.33)	<0.001 (1.21)	2.98 ± 2.01 (2.44–3.52)	1.52 ± 2.74 (0.12–2.93)	0.012 (0.64)
2nd year (2018)	5.24 ± 1.53 (5.01–5.43)	2.70 ± 1.62 (2.34–3.09)	<0.001 (1.61)	5.35 ± 1.62 (4.92–5.79)	2.82 ± 1.13 (2.24–3.40)	<0.001 (1.81)
3rd year (pre pandemic 2019)	3.04 ± 1.39 (2,76–3.13)	1.41 ± 2.19 (0.90–1.89)	<0.001 (0.88)	3.07 ± 1.52 (2.66–3.47)	2.70 ± 2.91 (1.20–4.20)	0.428 (0.46)
Total ED visits	11.17 ± 3.41 (10.58–11.48)	5.11 ± 3.46 (4.31–5.92)	<0.001 (1.76)	11.41 ± 3.75 (10.40–12.41)	7.05 ± 5.62 (4.16–9.95)	0.010 (0.91)

### 3.7. Mortality

**Figure 4:**
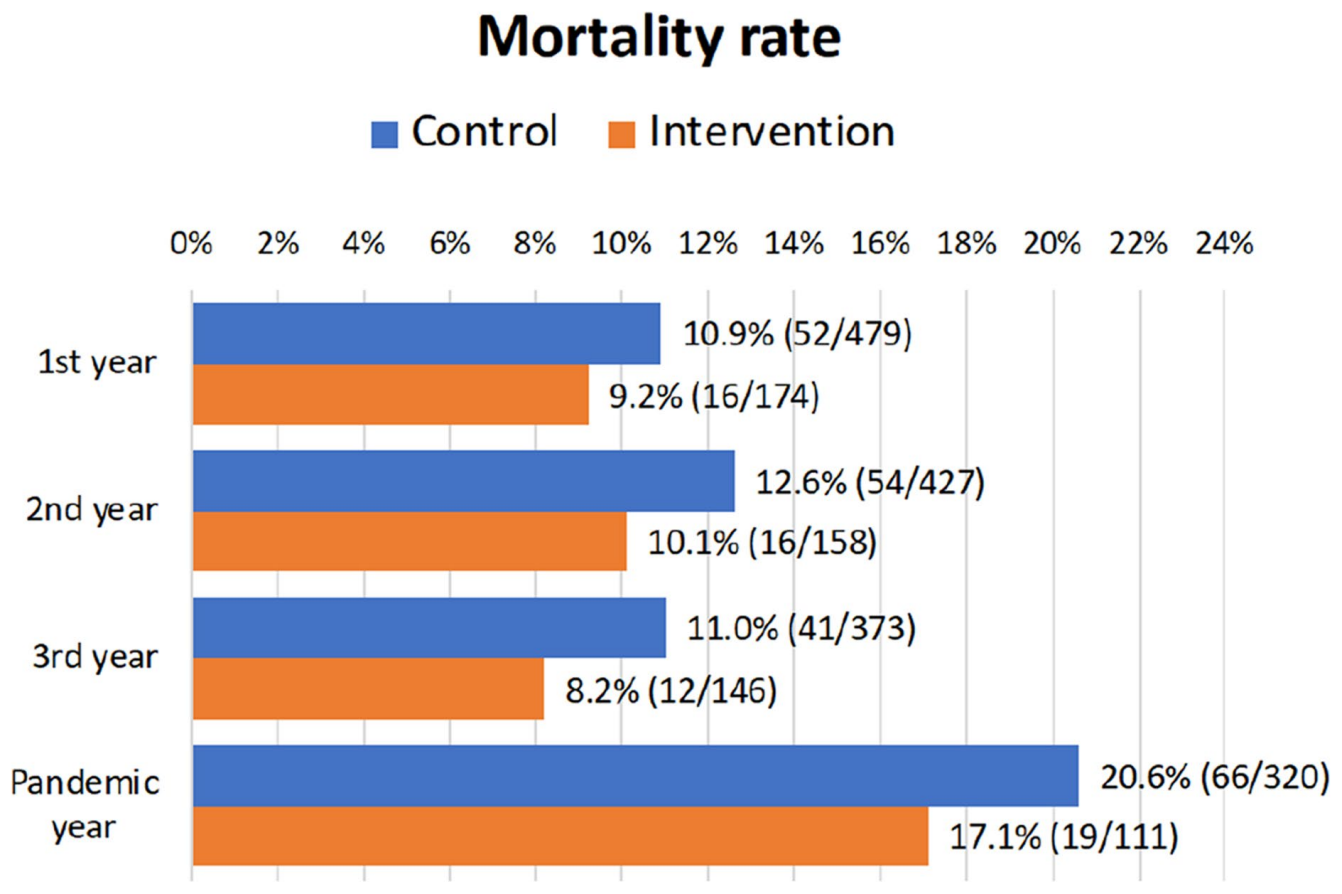
Mortality rate during the 3 years of nursing home intervention. Mortality rates during the pandemic year 2020 post-intervention are also shown.

During the 3 years of the study, 188 patients died (144 in the CG and 44 in the IG). These figures represent a mean of 9.6% per year of deaths in the entire sample. Figure 4 shows the mortality rate during the years of the nursing home intervention. The 3-year overall mortality rate was 30.6% in the control group and 25.3% in the nursing home visit program. These differences were not statistically significant (*p* = 0.274). At the time of analyzing the retrospective data in January 2021, other 85 participants died during 2020, the first year of the pandemic (66 in the control group and 19 in the intervention group), representing a mortality rate of 20.6% in the control group and 17.1% in the intervention group (*p* = 0.459). Therefore, the nurse-home visits program had no impact on the mortality rate.

## 4. Discussion

This study evaluated the efficacy of a nursing intervention program on a group of complex chronic patients and the residual effects of this intervention on patients for up to 3 years after implementation. The impact of the intervention on patients’ health was assessed by analyzing health resource utilization as measured by hospital admissions, emergency care, and home visits by primary care nurses. The global intervention was based on the following pillars: person-centered care, proactive recruitment by nurses of reference, and planning a minimum of three home visits per year. In this home visit, a holistic and comprehensive assessment of patients was performed, analyzing the degree of independence for daily living activities, cognitive impairment, and social support, together with knowledge about their nursing, therapeutic adherence, medication reconciliation, anticipation of decompensation, mobilizing resources early, establishing telephone contact 48 h post-admission with patients, identifying potential risks (i.e., falls, appearance of pressure scars), and adopting preventive measures.

The intervention program demonstrated its effectiveness in a substantial reduction of hospital admissions and visits to the emergency department. These findings are clinically relevant particularly in two aspects. First, healthcare management of CCP can be carried out by properly trained nurses providing high-quality care that decreases the needs of primary care physicians (30) and second, nursing home visits contribute to save costs by reducing the utilization of expensive health resources. Our study substantially reinforces the role of primary or community care nurses and advocates for increasing resources to incorporate new nursing services and acquire new competencies to respond to the challenges that primary care face today.

According to the results, both the intervention and control groups have similar characteristics, with a comparable distribution in terms of sex, average age, and the profile of complex chronic patients described in previous studies (31–33). One of the main effects observed in the intervention group was a significant decrease in hospital admissions, particularly during the two first periods of the study. The current results are comparable to previous investigations, where a lower number of hospital stays was also observed during the first year of program implementation and a moderate reduction of this effect in patients after the second year (32). This effect on the reduction of hospital admissions and visits to the emergency department could be due to the more exhaustive follow-up by primary care nurses, who would prevent patients from arriving at the hospital with more advanced degrees of decompensation by identifying and treating exacerbations earlier.

One of the most significant practical impacts of reducing hospital admissions and ED visits is cost savings. Hospital care is expensive, and a significant portion of healthcare spending is attributed to inpatient care and emergency services. In some cases, hospital admissions and ED visits may result from overutilization of healthcare services. By reducing hospital admissions and ED visits, healthcare facilities can allocate their resources more efficiently. This includes staff, beds, equipment, and supplies. When resources are used more effectively, healthcare organizations can operate more cost-effectively with substantial cost reductions. All these economic factors derived from the decrease in hospital admissions and ED visits were not analyzed in our study. However, our results emphasize the importance of primary care nurses’ role in managing CCP, especially in reducing healthcare resource utilization and consequently reducing expenditures (33).

Data from a recently synthesized analysis of several systematic reviews (the umbrella review), including a large population of older people (>60 years), demonstrated a small favorable effect of home-visit nursing on reducing the number of hospital admissions, but no quality of life and mortality rate (34). Most of the studies covered by the umbrella review did not include a clear description of the content of home visits, qualification of nurses, intensity of intervention, or follow-up intervals. The limited benefit concerning the reduction of hospital admissions was not related to the profile and frequency of home visits or follow-up intervals. Furthermore, in many studies, there were important differences in compliance with the intervention, and there was a lack of information concerning the usual care received by the comparison groups.

According to the authors of the umbrella review, the limited effectiveness of home-visit nursing in reducing hospital admissions may be attributed to a combination of factors with opposing impacts. First, the increase in admissions of older individuals who needed hospital or institutional care, but were previously overlooked, could have contributed to this outcome. On the other hand, some admissions might have been prevented through home visits, which could explain the positive effect of reducing hospital admissions (35). Additionally, variations in admission policies across different countries might also play a role in the lack of significant impact of home visit nursing on admission rates to hospitals and long-term care institutions. These policy differences may lead to varying outcomes in different regions and could potentially obscure the overall effectiveness of home-visit nursing in reducing hospital admissions.

Some reviews have indicated that the effectiveness of nursing home interventions might be more pronounced for older individuals with poorer health, younger study populations, or individuals at a lower risk of death (21). However, the specific reasons for these discrepancies among reviews regarding factors influencing intervention effectiveness remain unclear and warrant further investigation. In the current study, the effect of the intervention was more evident among participants with null or low disability status than among those with moderate or high disability status.

Significant results were also obtained in terms of the number of hospital emergency department visits in the 3 years after program implementation. Patients in the intervention group visited hospital emergency departments less than those in the control group. This effect could also be attributable to better control by their nurse of reference and to the knowledge acquired regarding the self-management of their underlying pathology, favoring the self-efficacy of the patients and/or main caregivers in the self-management of their disease (36). In this sense, the results obtained are also similar to the conclusions reached by other studies carried out in similar settings but in different countries reporting a reduction in the number of emergency room visits and hospital admissions (37, 38).

It is true that when relating emergency department visits to the degree of dependence of the patients, the figures between the control group and the intervention group for visits to the emergency department of the most dependent patients tended to equalize from the second period onwards. This could be explained by the passage of time and the increase in fragility and dependence of these patients, which in turn is related to the increase in morbimortality (21, 39, 40). The current findings support the need to reinforce preventive interventions much more and, therefore, emphasize the importance of Primary Care nurses with advanced competencies in chronicity, particularly in the group with more independent CCP. Furthermore, it seems relevant to reinforce more technical and clinical interventions to avoid decompensation in dependent patients.

Regarding the influence of the home visits program on the mortality rate, the lack of effect in our intervention aligns with findings from two extensive systematic reviews and meta-analyzes (4, 34). In the first review, which included 53 studies with over 23,000 participants, home visiting did not consistently show an association with differences in mortality. There were no significant variations observed among subgroups when studies were stratified by the focus of intervention, average age, or number of visits. Ten studies, comprising a total of 2,563 control participants without interventions and 2,491 home-visited patients, reported mortality rates at 3-year follow-up, that is the period covered by our series. Notably, there were no differences in mortality between the two groups (risk ratio = 0.82 [0.66, 1.00], Chi2 = 1.29, df = 9, *p* = 0.15; I2 = 32%). The mortality rate (11.5% in the control group and 9.5% in the home visits group) was lower than that observed in our study, although most of the included series did not report the prevalence of complex chronic patients.

In a more recent comprehensive review of the impact of home visit nursing on mortality, nine systematic reviews (4, 13, 22, 23, 35, 41–44) integrated data from 20 relevant randomized controlled trials, encompassing a total of 10,455 participants (34). Notably, only one old Danish trial (45) with 572 participants, aged 75 years or older and residing in a suburb of a major Nordic city, reported a lower mortality rate at the 3-year follow-up for patients receiving home visits compared to those receiving standard care or no intervention (19.6% versus 26.1%, *p* < 0.05). However, all other trials did not demonstrate a significant impact on mortality. When considering all these trials collectively, factors such as the nature of home visits, the intensity of interventions, and the duration of follow-up did not appear to influence mortality. In summary, our results agree with the cumulative evidence suggesting that home visit nursing had no substantial effect on mortality. In fact, the decrease in mortality in these chronic patients was not included in the main objectives of the program.

A limitation of the present study concerns to its retrospective nature. Conducting a randomized clinical trial was unfeasible due to the specific population being studied (CCPs) and the limited human resources available to deliver the desired services. This study involves a comparison between the traditional approach for managing CCPs in a specific Primary Healthcare area and an innovative healthcare model involving standardized nurse home visits in the same area. It is noteworthy that both groups exhibited no significant differences in comorbidity and dependency levels at the baseline, thereby enhancing the importance of the obtained results. Like other quasi-experimental studies, it is important to acknowledge the potential introduction of a Hawthorne effect, where participants may modify their behavior when aware of being observed. Regrettably, this is an inherent factor that cannot be eliminated.

Another limitation of the study lies in the absence of an evaluation of the effectiveness of home visit nursing in terms of other relevant outcomes, such as patient satisfaction, quality of life, the duration of hospital admissions, and the location or cause of death. In addition, it was not possible to independently assess the impact of the three stages that constitute the nurse home visits on patient health outcomes. Finally, although it is true that an economic evaluation of the program has not been carried out–which is one of the limitations of our study–it can be assured that while in specialized care, the program resulted in savings, in primary care, the costs would have increased due to the increase in home visits. The difference has been found to be cost-effective when related to avoided hospital admissions (33, 46).

Future research is still necessary to explore the optimal intensity of home visits and identify specific populations that can benefit the most from this approach. A comprehensive and detailed description of the care delivery process, including intervention compliance, qualifications and training of care providers, and standard care received by comparison groups, is essential. Such reporting will provide valuable insights into elements that may be beneficial to home-visit programs. Countries that have integrated home-visit services into their national healthcare policies should carefully assess the merits of these services, considering their local healthcare system objectives and contextual factors. Adopting a thoughtful and context-specific approach will ensure that the implementation of home-visit programs aligns with the unique healthcare needs and goals of each region.

## 5. Conclusion

The comprehensive CCP program that was evaluated demonstrated its effectiveness in terms of reduced use of health services, particularly hospital admissions and visits to the emergency department. This study demonstrates the crucial and often underappreciated role of primary care nurses in managing complex chronic patients. Their contributions in coordinating care, educating patients, and promoting preventive measures can lead to substantial reduction of expensive healthcare resources. More studies are needed to analyze the true cost-effectiveness of these interventions in the primary care setting and to promote health policies that reinforce primary care.

## Data availability statement

The raw data supporting the conclusions of this article will be made available by the authors, without undue reservation.

## Ethics statement

The studies involving humans were approved by Ethics Committee for Clinical Research of the Department of Health, La Ribera, Valencian Community, Spain. The studies were conducted in accordance with the local legislation and institutional requirements. The participants provided their written informed consent to participate in this study.

## Author contributions

MSS: Conceptualization, Data curation, Methodology, Writing – original draft. JC-L: Methodology, Investigation, Writing – review & editing. CB: Writing – review & editing, Conceptualization, Formal analysis, Supervision.
